# Toward better understanding of the human placenta: development of “disease-in-a-dish” models

**DOI:** 10.1186/1471-2393-15-S1-A6

**Published:** 2015-04-15

**Authors:** Mana M Parast

**Affiliations:** 1 Department of Pathology and the Sanford Consortium for Regenerative Medicine, University of California San Diego, California, USA

## 

Many stillbirths result from pregnancy complications, whose root cause is abnormal development and function of the placenta [1]. In order to prevent stillbirths, we need to have a better understanding of how the human placenta develops, both in normal and abnormal pregnancies. This lack of understanding of the human placenta has recently been acknowledged, and “The Human Placenta Project” launched, by the National Institute of Child Health and Human Development (NICHD) [2]. In fact, the human placenta is difficult to study because of the lack of both “in vivo” animal models and placental cell lines able to be cultured “in vitro” in a tissue culture dish. Specifically, mice and rats have placentas which differ from the human both in structure and at the molecular level [3]; in addition, the human placental cell lines behave differently in culture, compared to the placental cells as they exist “in vivo” in the pregnant patient [4]. Over the last 5 years, our laboratory has set out to use human pluripotent stem cells (hPSCs) to model placental development in a dish [5]. “Pluripotent” stem cells have the ability to differentiate, or turn into, any cell type in the body, including the placental cell type, “trophoblast”[6,7]. While initially hPSCs had to be derived from human embryos, in 2007, Yamanaka et al. developed a method for generating such cells from any proliferative cell type [8]. hPSCs have now been derived from numerous cell types, including amnion cells of the placenta [9].

We have developed a method for step-wise differentiation of such hPSCs, first into trophoblast precursor cells and then into terminally differentiated, functional trophoblast, including multinucleated syncytiotrophoblast (STB) and invasive extravillous trophoblast (EVT). These two cell types are the functional units of the placenta: STB carry out nutrient and gas exchange, while the EVT invade the maternal uterus and establish blood flow to the feto-placental unit. Our differentiation method is both reproducible and highly efficient, with >95% of cells becoming trophoblast in the culture dish, based both on expression of specific genes and on functional assays such as secretion of the pregnancy hormone, hCG. We recently applied this method to hPSCs carrying a chromosomal aneuploidy, Trisomy 21 (T21). It is known that trophoblast isolated from T21 placentas have a defect in differentiation into multinucleated, hCG-secreting STB [10]. We asked whether this defect could be reproduced in culture when differentiating T21 hPSCs into trophoblast. We observed that T21 hPSCs indeed show delayed differentiation into functional STB, secreting significantly less hCG into the media compared to trophoblast derived from hPSCs with a normal karyotype. These results confirm the utility of hPSCs in modeling human placenta, both during normal development and in disease. We are currently collecting and banking amnion epithelial cells from placentas of patients with pregnancy complications, focusing on early-onset severe preeclampsia, which is highly associated with both maternal and neonatal morbidity and mortality. We believe that, once reprogrammed into hPSCs, these cells hold great promise, both in advancing our understanding of the mechanisms of placental dysfunction, and also in providing a platform for drug screening to reverse the disease phenotype.
